# Phase Coexistence and Kinetic Arrest in the Magnetostructural Transition of the Ordered Alloy FeRh

**DOI:** 10.1038/s41598-018-20101-0

**Published:** 2018-01-29

**Authors:** David J. Keavney, Yongseong Choi, Martin V. Holt, Vojtěch Uhlíř, Dario Arena, Eric E. Fullerton, Philip J. Ryan, Jong-Woo Kim

**Affiliations:** 1 X-ray Science Division, Argonne National Laboratory, Argonne, IL 60439 USA; 2 Center for Nano-Materials, Argonne National Laboratory, Argonne, IL 60439 USA; 3 Center for Memory and Recording Research, University of California San Diego, La Jolla, CA 92093-0401 USA; 4 Department of Physics, University of South Florida, Tampa, FL 33620 USA

## Abstract

In materials where two or more ordering degrees of freedom are closely matched in their free energies, coupling between them, or multiferroic behavior can occur. These phenomena can produce a very rich phase behavior, as well as emergent phases that offer useful properties and opportunities to reveal novel phenomena in phase transitions. The ordered alloy FeRh undergoes an antiferromagnetic to ferromagnetic phase transition at ~375 K, which illustrates the interplay between structural and magnetic order mediated by a delicate energy balance between two configurations. We have examined this transition using a combination of high-resolution x-ray structural and magnetic imaging and comprehensive x-ray magnetic circular dichroism spectroscopy. We find that the transition proceeds via a defect-driven domain nucleation and growth mechanism, with significant return point memory in both the structural and magnetic domain configurations. The domains show evidence of inhibited growth after nucleation, resulting in a quasi-2^nd^ order temperature behavior.

## Introduction

Magneto-structural phase transitions offer new ways of accessing multiferroic properties, as well as a probe of the relationships between structure, magnetic and electronic ordering^1^. The phenomenology of these coupled transitions is immensely varied, as they may arise in any condensed matter system where structural, magnetic and electronic energies are closely matched. This can result in large changes of electronic/magnetic order with very subtle structural transitions. Often, materials that display such multiferroic behaviors have complex crystalline structures, with multiple avenues for distortions and symmetry changes that may result in electronic property modifications. While these materials offer much promise for discovery of new phases and applications, systems that exhibit coupled transitions with a relatively simple structural change may enable better control of the transition as well as the opportunity to study the physics of the coupling.

The CsCl-structured alloy FeRh experiences a first-order structural phase transition above room temperature characterized by a ~1% isotropic volume expansion coupled with a magnetic transition from a low-temperature antiferromagnetic (AFM) phase to a high-temperature ferromagnetic (FM) phase^2–10^. During the magnetic transition, the Fe sublattice switches from antiparallel to parallel exchange, while the Rh develops a magnetic moment of approximately 0.9 µ_B_^11–14^. The transition temperature can be tuned easily by an applied magnetic field, with a reported effect of −9 K/T^15,16^. In addition, other external stimuli, such as piezoelectric strain has been found to influence the magnetization of the FM phase^9^ as well as the magnetocaloric properties^17^ near the transition. In epitaxial films, the isotropic expansion is modified by substrate clamping to become an out-of-plane tetragonal distortion^18^. The transition temperature may vary in films with thickness and substrate mismatch playing a role, however the magnetic phase transition still remains coupled to the structural change as in the bulk counterpart. Therefore, because epitaxial FeRh films display an intrinsic coupling between the structural and magnetic degrees of freedom, but in a relatively simple system without a change in symmetry, the system represents an opportunity to probe the fundamental nature of magneto-structural coupling in a straightforward way. This material is also of interest for magnetic storage applications, due to the potential to use the high-temperature FM phase as a write-assist layer for heat-assisted magnetic recording or the AFM phase for memory elements.

In addition to the macroscopic properties of this material, the evolution of domains is important for understanding how the transition proceeds, especially for applications which rely on fast switching between states or mesoscale sizes. The morphology of the phase transition can also shed light on the dynamics in first order phase transitions in general. Recently, spatially resolved studies of the magnetic transition using X-ray photoemission electron microscopy (X-PEEM) have been reported, in which a domain growth and nucleation process were observed^9,19,20^. However, studies of the structural domain evolution, and especially coupled magnetic and structural experiments have been less common, owing to a lack of real space probes of lattice parameter with adequate spatial resolution.

Here, we apply multimodal nanoscale imaging to examine the domain morphology in both the structural and magnetic transitions, coupled with wide area x-ray magnetic circular dichroism (XMCD) spectroscopy to track the correlation between the structure and the Rh and Fe moments in epitaxial FeRh(001) films. In our imaging studies we use nano-x-ray diffraction (XRD) with a spatial resolution of ~30 nm and X-PEEM with a spatial resolution of ~100 nm. We find that the structural transition proceeds via a nucleated domain growth mechanism, likely initiated by localized modifications of the energy landscape near defects, with significant regions of phase coexistence prior to and after percolation. The magnetic transition mirrors the nucleation and growth model, with large final domains in the high temperature phase resulting from coalescence.

## Results

**Figure 1:**
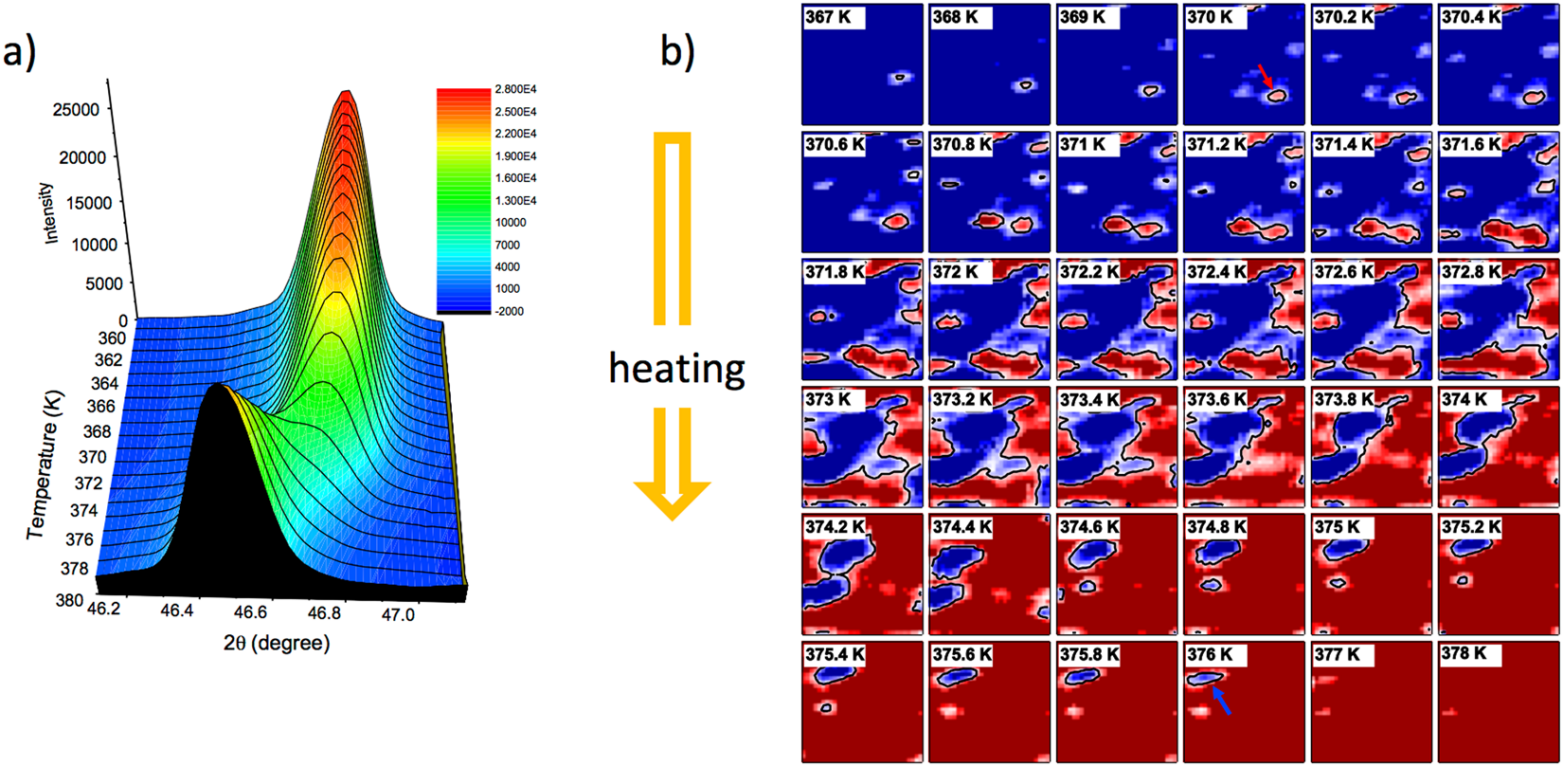
(**a**) Temperature dependent XRD data taken at the FeRh(002) reflection for the heating transition at 375 K. (**b**) Corresponding temperature dependent nano-XRD data taken with a 30 × 30 nm^2^ focused spot. Red (blue) indicates the low (high) temperature phase of FeRh.

In Fig. 1, we show the zero-field structural phase transition during heating as indicated by the nano-XRD results. Figure 1a shows the evolution of the average lattice expansion through the AFM-FM phase transition. Figure 1b shows the spatially resolved diffraction results during heating through the phase transition which clearly show the heterogeneous mixture of the AFM and FM phases during the transition. Here, we note the evolution of the high-temperature FM phase as nucleated islands that are static on the time scale of the measurement (several minutes to hours). As the initial islands grow with increasing temperature, new ones nucleate at higher temperatures, which then grow to join the larger islands. This suggests defect-driven nucleation, in which the material surrounding a defect has a lower effective transition temperature due to localized strain and/or electronic structure modifications. In this case a distribution of nucleation temperatures would arise from defects of different types. We note that this proposed mechanism is opposite to pinning, in which a defect site provides a local energetic barrier to the growth of a transition.

**Figure 2:**
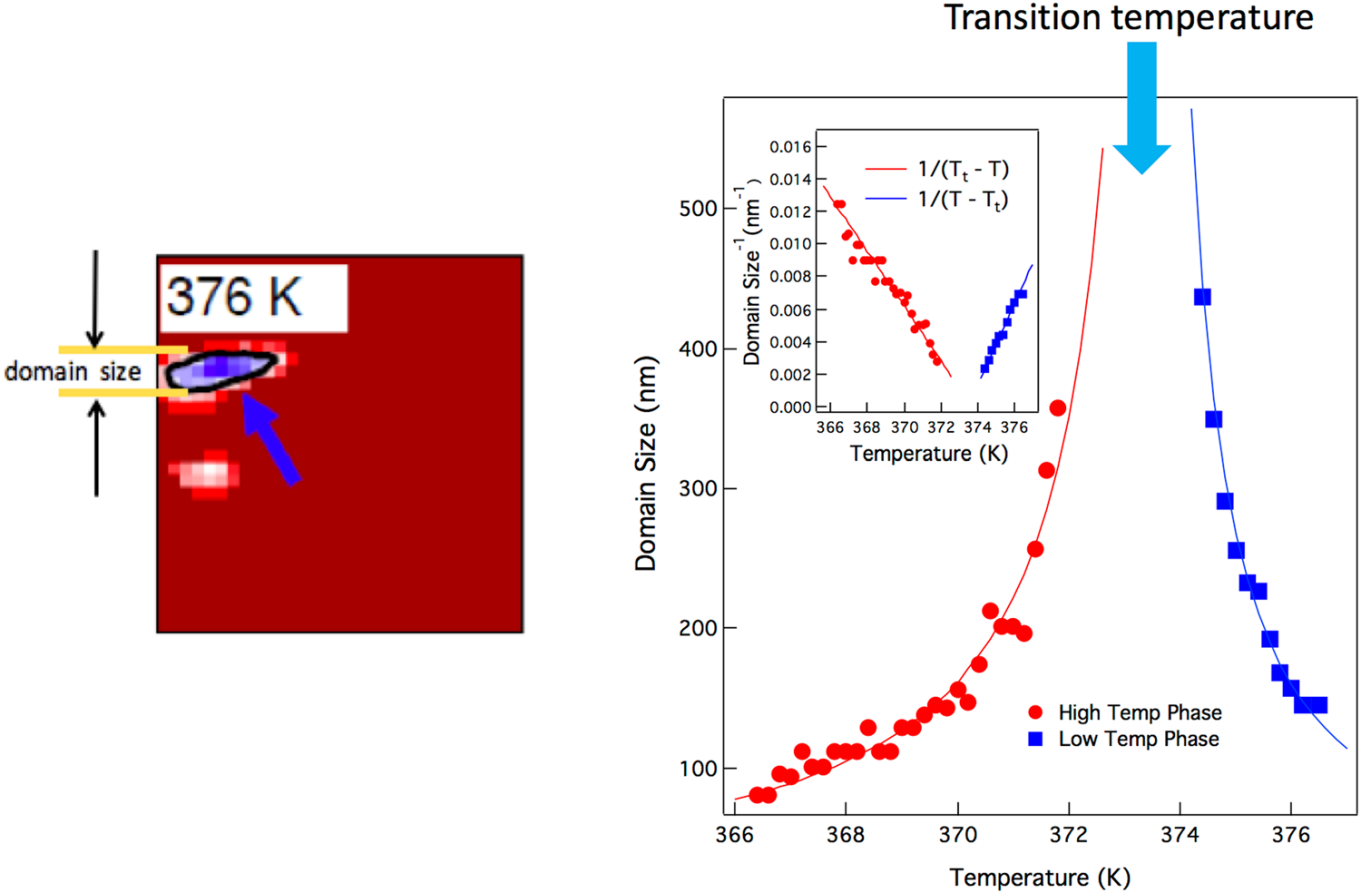
Temperature dependence of the island size for a selected nucleated island taken from the image data in Fig. 1. All data are for the heating transition. In both the nucleation and growth of the high temperature phase islands below the transition and the shrinking of the remaining low temperature phase islands above it, we observe a 1/T – like power law, indicating stable islands whose size depends only on temperature. Inset: Inverse domain size vs T, showing linear dependence.

The subsequent growth of each island is apparently restricted by the surrounding material, which implies that material not neighboring a defect has an elevated transition temperature. Therefore, the energetics of the transition are such that even in the proximity of FeRh in the FM phase, the AFM phase is still stable just below its transition temperature. This kinetic arrest of the domain growth results in a temperature dependence of the domain size that deviates from the expected behavior for a 1^st^-order phase transition, as we show in Fig. 2. Here, we follow the size of a single domain derived from the nano-XRD images in both the FM and AFM phases through the phase transition. For both the increase of FM domain size below the transition and the decrease of AFM domain size above it, we see an inverse relation with the quantity |(T - T_t_)|, where T_t_ is the transition temperature. This scaling phenomenon is usually associated with the critical behavior of a 2^nd^ order transition with a correlation length scale of several hundred nanometers. Large-scale correlation lengths have been observed in heterogeneous nucleation processes with both 2^nd^ and weak 1^st^-order transitions. In these cases, elastic deformation from defect centers can generate strain fields that extend over large distances, creating large-scale correlations prior to 2^nd^-order transitions^21–23^. Similar kinetic arrest was reported in annealed polycrystalline ingots of Pd-doped FeRh alloy^24^ and interpreted as the formation of a glass-like intermediate state. We also note that a recent study of the metal-to-insulator transition in the highly correlated system V_2_O_3_ showed a similar temperature dependence of the domain size that was attributed to a competition between short and long-range ordering interactions^25^. Here, the observed long-range critical behavior is extraordinary in this case of a strong 1^st^ order solid-solid transition, and we interpret it in terms of a competition between energetically favorable strain states.

**Figure 3:**
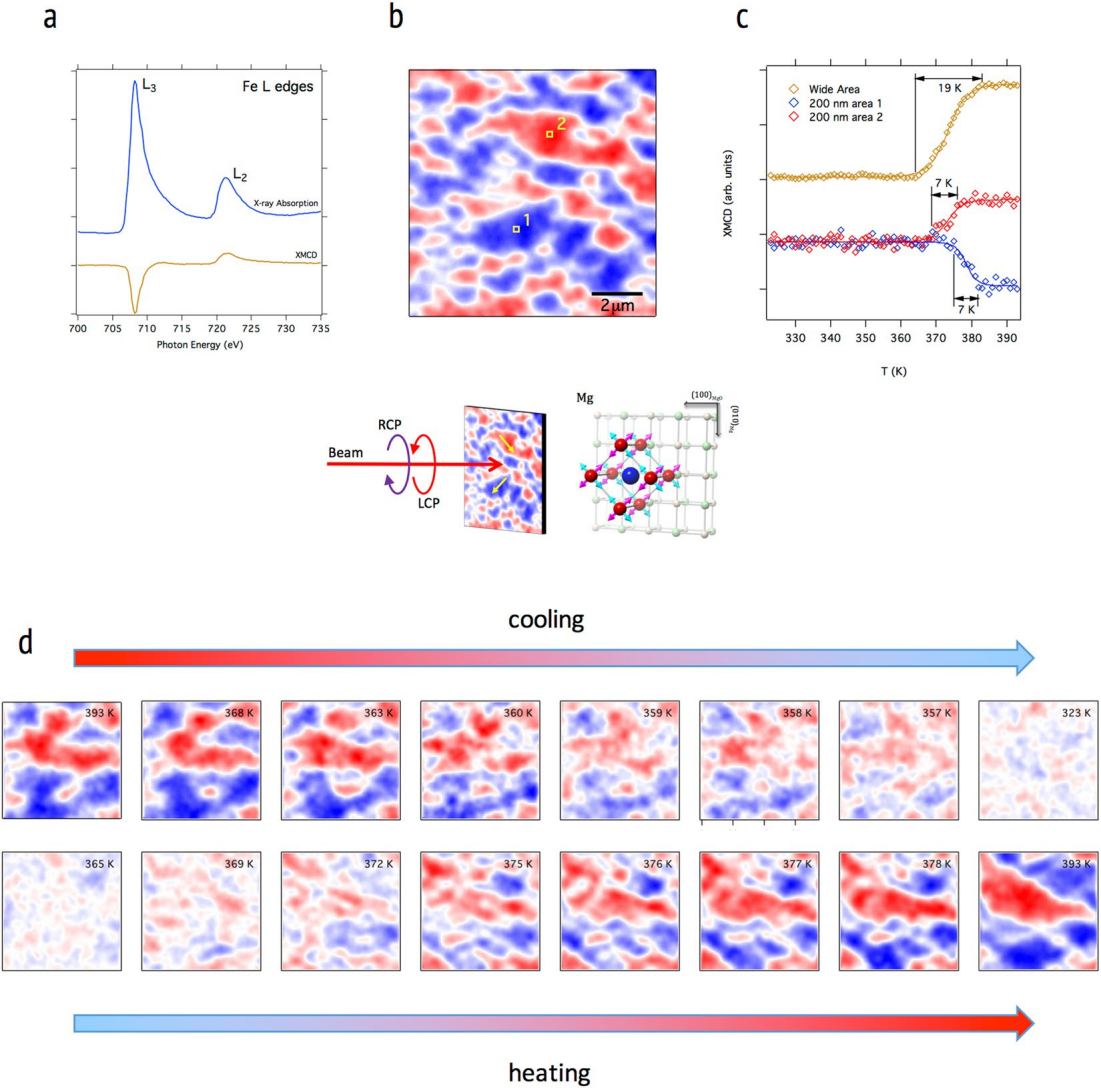
Temperature dependent X-PEEM data showing the magnetic domain evolution during the phase transition. By tuning the incident photon energy to an appropriate resonance, the strong XMCD effect is exploited, as in (**a**), to obtain magnetic domain images from the difference of LCP and RCP images (**b**). Data in (**a**) and (**b**) were taken at 410 K. In (**c**), we show the magnetization during the heating transition for the local domain magnetization (red and blue) and the average magnetic ordering over a wide area. The local magnetization plots are derived from the pixel sums of two 200 × 200 nm ROIs as shown by the yellow squares in (**b**). The wide-area plot is derived from the standard deviation of the pixel sum over the entire image in (**b**), giving a measure of the magnitude of the domain magnetizations along the beam direction independent of their signs. Open symbols in (**c**) are experimental data, and the corresponding lines are Brillouin fits. We note that the single domain transitions are narrower than the average, and have varying initial nucleation and transition midpoint temperatures. The transition midpoint temperature, T_0_, for areas 1 and 2 are 378.5 and 373.2 K, respectively, while for the average transition T_0_ = 373.4 K. This supports a model of the transition in which defects lower the transition temperature via local strain, resulting in a distribution of transition temperatures that broadens the overall transition. In (**d**), we show a detail of the domain nucleation and growth through a complete cooling (upper) and heating (lower) cycle in 4 × 4 μm temperature dependent X-PEEM images.

To examine the magnetic transition, we show in Fig. 3 the temperature dependent X-PEEM images taken at the Fe L_3_ x-ray absorption edge. In this data, we are sensitive only to the net FM moment along the beam direction, therefore the low temperature AFM phase appears uniformly neutral white, while in the high-temperature FM phase red and blue domains are evident corresponding to parallel and antiparallel to the x-rays. In the evolution of these domains, we see a similar nucleation and growth process as observed in the structural domains, including a distribution of nucleation temperatures closely clustered around the nominal transition point. This is demonstrated in Fig. 3c, where we show the evolution of FM order on heating of a wide area covering many domains, and of two small (200 nm × 200 nm) regions. We see that the two small regions both have a ~7 K width to the FM transition, and we note that this is true of six other similar regions that are not shown. However, the transition temperatures, defined by the midpoint of the transition, vary by several Kelvins. We also note that the wide area plot shows a 19 K width, which is consistent with a sum of multiple narrower transitions with varying transition temperatures. The magnetic domains then coalesce as the transition progresses. The final magnetic configuration is characterized by a larger domain size than the initial structural domains, and is stable up to 420 K.

**Figure 4:**
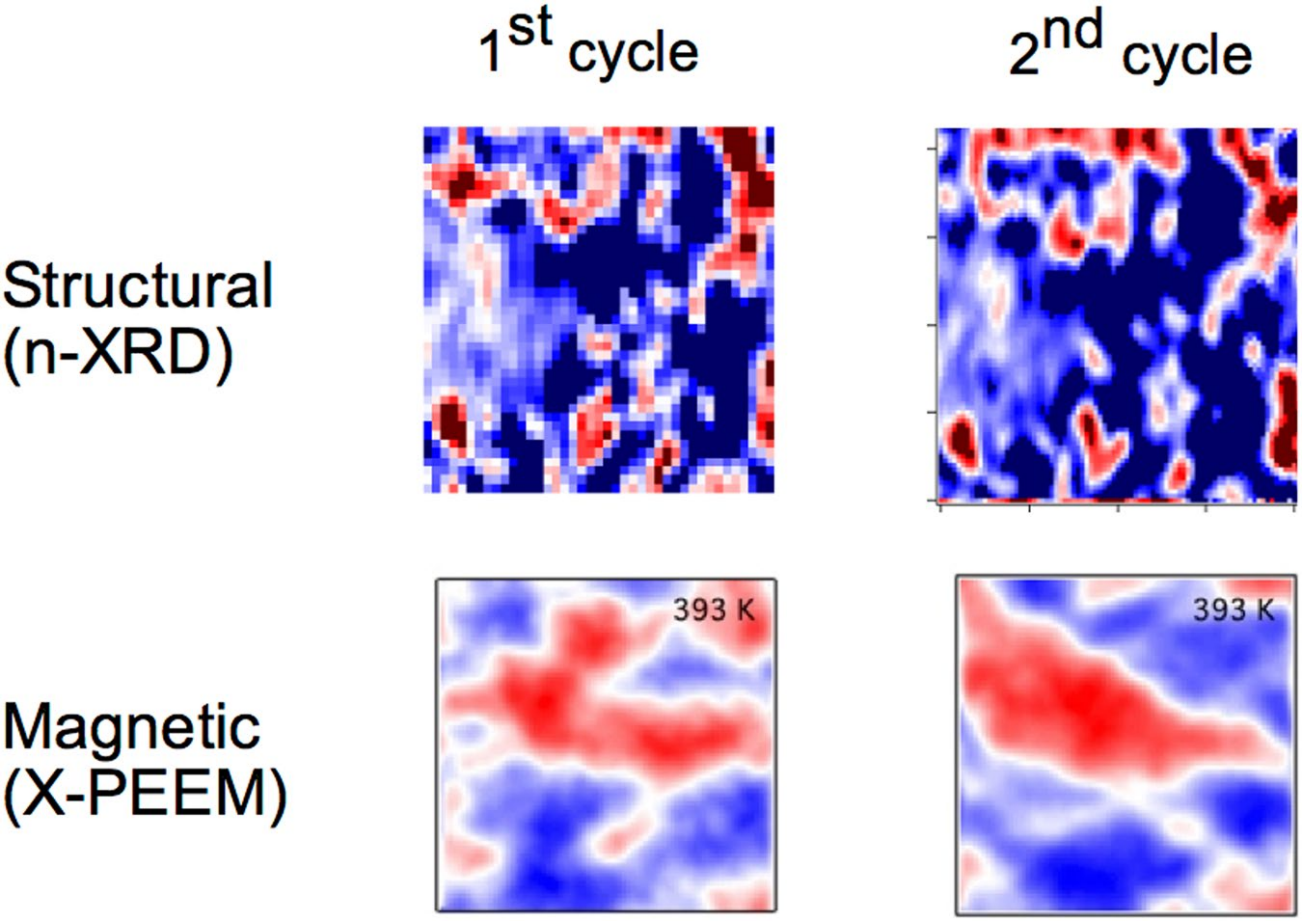
Nano-XRD images taken at the midpoint of the structural transition (upper) and X-PEEM images taken in the high temperature phase (lower) for two consecutive heating and cooling cycles. Note that the structural and magnetic images show two different contrast mechanisms. The structural data show the formation of high temperature phase islands in a matrix of low-temperature phase, while the magnetic data indicated domains of opposite magnetization in the high-temperature phase. In the structural transition, the return point memory is nearly complete, with a close correlation between the domain configurations from cycle to cycle. The magnetic transition displays more stochasticity in the final domain states, although some return point memory does exist. All images are 4 × 4 μm.

In Fig. 4, we demonstrate the return point memory characteristics, or the correlation of specific domain configurations from one hysteretic cycle to the next, of both the structural and magnetic components of the transition. Specifically, the configurations of the structural domains at the midpoint of the transition, and the magnetic domains in the fully FM phase, after the transition for consecutive cycles are shown. We note that the return point memory of the structural transition is nearly complete, with a domain-by-domain correlation between cycles. This strongly supports the proposed defect-driven domain nucleation model of the transition. Here, defects locally modify the symmetry or electronic structure of the FeRh material surrounding them, thus lowering the local transition temperature and allowing droplets of phase changed material to form around them. Since these defects are static, their locations are frozen into the material, thus the locations of the initial nuclei would be expected to be the same with subsequent cycling. The magnetic transition’s return point memory is less complete. Here, there is a general reproduction of the location of large-scale domains, but their shapes, locations or even existence of smaller domains may change from cycle to cycle. This suggests that while the defects may govern the initial formation of the FM domains via the formation of isolated islands of FM phase, once percolation is achieved the defects do not effectively pin the magnetic configuration. This is consistent with the relatively low coercive field in the FM phase suggesting a relatively low pinning of domain walls. Thus, while the magnetic configuration is stable on the order of the observation time (several minutes), magnetic domains coalesce once they are able to interact with each other.

## Discussion

**Figure 5:**
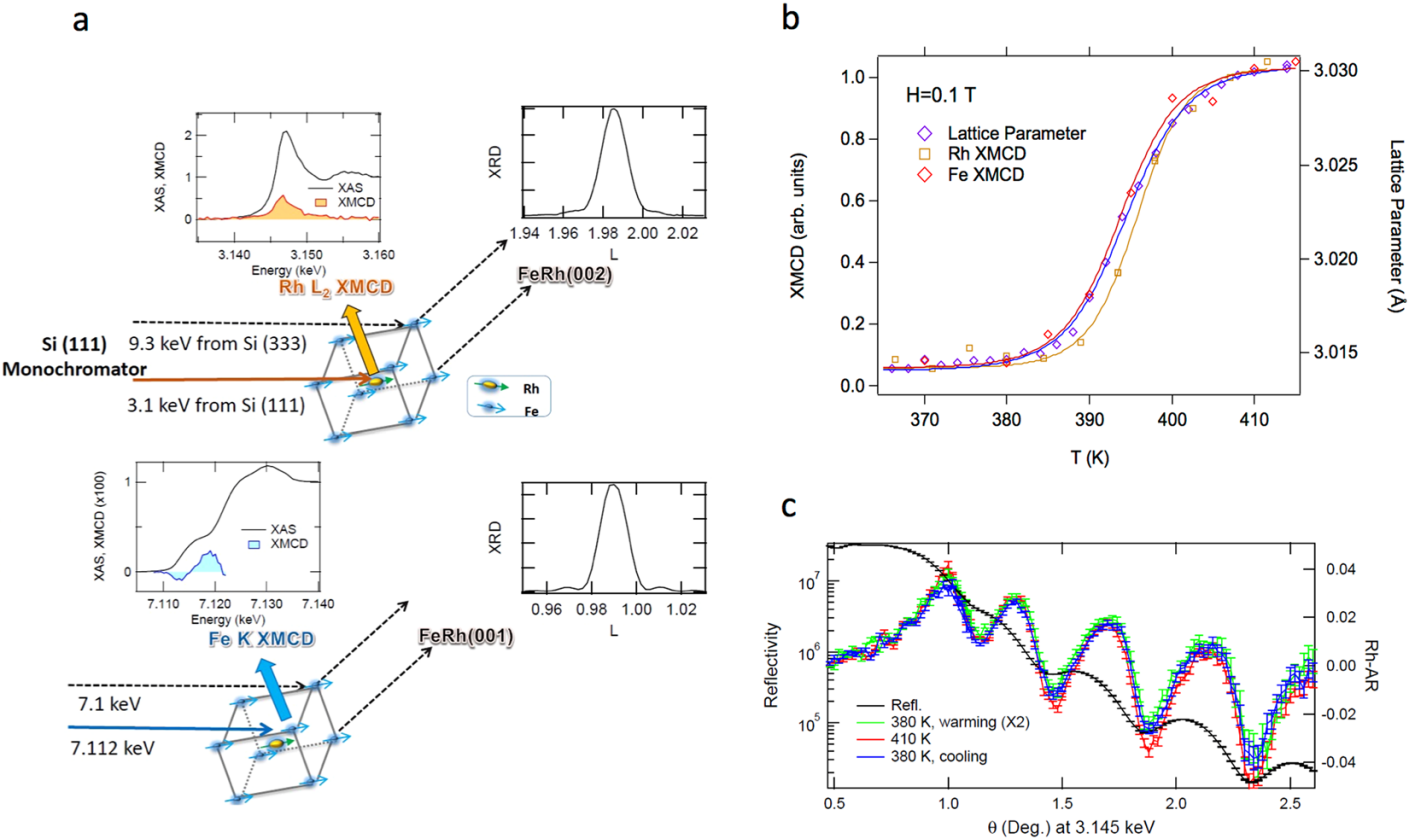
Concurrent lattice parameter and magnetic measurements at Fe and Rh during the heating transition. Data were collected using XMCD and diffraction in a single experiment with common thermometry as shown in (**a**) and described in Methods. (**b**) Comparison of the Fe K and Rh L_2_ XMCD spectroscopy with FeRh lattice parameter. For Fe and Rh XMCD curves, integrated areas of the Fe K and Rh L_2_ XMCD spectra are plotted as a function of temperature. The temperature dependent lattice parameter was calculated from the FeRh (002) reflection. The lattice parameter from FeRh (001) curve was equivalent to the (002) and thus not shown. Open symbols are data and colored lines are Brillouin function fits to the corresponding data. The transition temperatures derived from the fits for the structure, Fe K XMCD, and Rh L_2_ XMCD are T_0_ = 394.4 ± 0.14, 393.7 ± 0.53, and 395.7 ± 0.33 K, respectively, where the uncertainties given are the error estimates from the least squares fitting. (**c**) Asymmetry ratio (AR) in magnetic reflectivity curves near the Rh L_2_ edge were measured during warming and cooling cycles as well as the maximum measurement temperature. The line-shape in AR is sensitive to the depth profile of Rh moment. At these temperatures, asymmetry ratio simply changes by a scaling factor without any significant change in line shape. This indicates that the phase domain growth occurs primarily in the plane of the film while in the surface normal direction the transition happens uniformly. The similar magnetic depth profiles support that the observed temperature evolution is not due to surface/interfacial effects.

While the structural and magnetic imaging show very clear correlations in the morphological evolution between magnetic and structural domains, by necessity the two studies have been carried out at separate facilities with differences in thermometry. Therefore, whether or not the domain nucleation temperatures are exactly correlated is still not known. To this end, we have examined the evolution of the average structural and magnetic order employing combined XRD with Fe K, and Rh L_2_ edge XMCD spectroscopy in which the same experimental set up is used, providing common thermometry (see Methods). In Fig. 5, we show the temperature dependence of the Fe K and Rh L_2_ XMCD spectroscopy along with lattice parameter data in an applied field sufficient to saturate the ferromagnetic phase (0.1 T). Fits to Brillouin functions show that the transition temperatures of the structure, Fe XMCD, and Rh XMCD are 394.4 ± 0.14, 393.7 ± 0.53, and 395.7 ± 0.33 K, respectively, where the uncertainties given are the error estimates from the least squares fitting. This shows that the structural transition coincides with the development of both the Fe and Rh moments to within 2 K, and supports the strong coupling between the structural and magnetic transitions observed in the spatially resolved XRD and PEEM imaging results. We also note that XMCD in x-ray reflectivity data (Fig. 5c) show that the magnetic depth profile does not vary except in magnitude of the moment as the film is cycled through the transition. This implies that the domain nucleation and growth process is uniform across the film depth, and does not involve a surface nucleation effect.

In summary, nano-scale structural and magnetic imaging of the magneto-structural AFM-FM phase transition in FeRh thin films shows a defect-driven domain nucleation behavior, with the growth of domains after nucleation arrested by the surrounding untransformed material. This results in a broad region of phase coexistence during the transition, and a 2^nd^-order-like temperature dependence of the domain size. Examination of individual magnetic domains reveals a uniform 7 K width to the transition in zero field, with variations in transition temperature that broaden the average width to 19 K. We see strong return point memory in the structural domain configuration, and some evidence for return point memory in the magnetic domains, which supports the defect-driven model of domain nucleation. Finally, an element and structure-resolved study with common thermometry confirms the coincidence of the structural transition and development of the Fe and Rh magnetic moments.

## Methods

### Sample Fabrication

Epitaxial 20-nm-thick FeRh(001) films were grown on MgO (001) substrates at 450 °C and an argon pressure of 1.5 mTorr by dc magnetron sputtering using an equiatomic target. The films were post-annealed at 850 °C for 2 hours, several of the samples were coated with 2nm of Ta. The crystallographic orientation is such that [100] direction of FeRh aligns with [110] direction of MgO (i.e. the FeRh crystal lattice is rotated by 45 degrees).

### Nanoscale Structural Imaging

Nanoscale X-ray Diffraction Microscopy experiments were performed using the Hard X-ray Nanoprobe (HXN) of the Center for Nanoscale Materials (CNM) at sector 26-ID-C of the Advanced Photon Source, Argonne National Laboratory. The monochromatic incident X-ray beam (photon energy 10.0 keV, *λ* = 1.2398 Å) was focused by a Fresnel zone plate yielding a ~30 nm beam size at the sample.

### Nanoscale Magnetic Imaging

Soft x-ray energy magnetic circular dichroic (XMCD) images were taken at the Advanced Photon Source beamline 4-ID-C using an Omicron photoemission electron microscope (PEEM). This instrument uses electrostatic electron optics to image the emitted secondary electrons from the FeRh surface with a typical spatial resolution of ~100 nm. The beamline uses a helical undulator and a spherical grating monochromator tuned to the Fe L_3_ resonance with a typical bandwidth of 0.05%. Difference XMCD images were obtained by acquiring separate exposures with LCP and RCP polarized radiation. The sample temperature was controlled using a filament mounted behind the sample and monitored with a thermocouple mounted on the sample holder.

### X-ray Diffraction and Spectroscopy with Common Thermometry

With common thermometry, combined diffraction and XMCD measurements were performed at the 4-ID-D beamline. A circularly polarized x-ray beam was generated with a diamond phase retarder. A magnetic field of ±0.1 T was applied parallel to the sample plane. XMCD spectra were recorded at the Fe K and Rh L_2_ edges in fluorescence mode using an energy dispersive detector. For the Rh XMCD and diffraction measurements were taken in one setting using with ~3.146 and 9.3 keV incident x-ray energies from the 1^st^ and 3^rd^ harmonic from the Si(111) monochromator crystal, respectively (Fig. 5). For the Fe K XMCD and diffraction measurements, the incident energies were ~7.112 and 7.1 keV, respectively.
